# Erythroid Cell Research: 3D Chromatin, Transcription Factors and Beyond

**DOI:** 10.3390/ijms23116149

**Published:** 2022-05-30

**Authors:** Charlotte Andrieu-Soler, Eric Soler

**Affiliations:** 1 IGMM, Université Montpellier, CNRS, 34093 Montpellier, France; charlotte.andrieu-soler@igmm.cnrs.fr; 2 Laboratory of Excellence GR-Ex, Université de Paris, 75015 Paris, France

**Keywords:** erythropoiesis, 3D genome organization, transcription factors, chromatin, enhancers, signaling, globin, thalassemia, anemia, leukemia

## Abstract

Studies of the regulatory networks and signals controlling erythropoiesis have brought important insights in several research fields of biology and have been a rich source of discoveries with far-reaching implications beyond erythroid cells biology. The aim of this review is to highlight key recent discoveries and show how studies of erythroid cells bring forward novel concepts and refine current models related to genome and 3D chromatin organization, signaling and disease, with broad interest in life sciences.

## 1. Introduction

Erythropoiesis is a complex and fascinating biological system. It stands out as being one of the largest cellular outputs of the human body, with the daily production of billons of new red blood cells (RBCs). It is also a remarkable system from a cell-biology perspective: erythroid cell production involves the precise coupling of cell cycle with cellular differentiation, culminating in massive intracellular organelle remodeling, nuclear condensation and expulsion, a process referred to as enucleation [1,2]. Despite the complete release of nuclear material, including genomic content, and cellular organelles such as mitochondria and ribosomal particles, mature erythrocytes show a remarkable robustness with structural flexibility and a life span of 120 days in the circulation.

These outstanding features of erythroid cells are to a large extent epigenetically programmed and controlled by the combinatorial action of numerous transcription factors (TFs), signaling pathways and metabolites, the interplay of which ensures the fine-tuning of the intricate differentiation steps leading to the production of mature RBCs. Erythropoiesis has been widely used as a model system to dissect the processes of cellular differentiation and to unravel gene regulatory principles. Several (epi)genetic concepts originate from findings originally obtained in erythroid cells, such as chromatin looping and dynamic enhancer–promoter communications, and the discovery of several key transcription factors, pathways and their intricate interplay. Erythroid cells still have wonders to deliver, and their study continues to be at the forefront of several research fields, providing new insights into basic science and human disorders. This review aims to summarize key recent discoveries, illustrating how the study of erythroid cells have generated tremendous functional insights into the fields of genomics, transcription factor dynamics and 3D chromatin organization, regulatory networks and signaling in healthy differentiation and disease.

## 2. Erythroid Epigenetics: Chromatin Landscape and 3D Chromosomal Contacts

### 2.1. Mammalian Genes and Distal Regulatory Elements

Erythropoiesis has long been used as a paradigm for cellular differentiation and disease. It has been an incredibly rich source of discoveries putting forward novel concepts over the past decades. An initial key observation stems from the analysis of globin genes, where it was found that mammalian gene transcriptional regulation is under the control of distal regulatory elements (e.g., enhancers) that can be located tens of kilobases away from promoters. The discovery of the globin locus control region (LCR) is one such example, and this peculiar feature was subsequently generalized, years after, with the development of high throughput chromatin profiling assays (such as ChIP-Seq, DNAse-Seq, and related assays thereof), which unraveled the complexity of the mammalian regulatory landscape [3,4,5].

Genome-scale profiling studies have shown that the number of gene regulatory elements such as enhancers largely exceeds the number of protein-coding genes, implying that genes are under the control of multiple enhancers. This is indeed the case for a large number of tissue-specific and house-keeping genes. For some well-studied loci, such as the *SHH* [6], *TAL1*, *RUNX1* [7,8,9] or *GATA2* [10,11,12] loci, which are under the control of multiple distal enhancers, each singular enhancer seems to provide selective tissue specificity and to orchestrate complex developmental expression patterns. However, this peculiar situation is not common, and many enhancers within a given locus were shown to possess some level of redundancy and to contribute to varying degrees to target gene expression in defined lineages and developmental windows. This is best exemplified by the α- and β-globin gene loci which are under the control of distal enhancer clusters that confer high level expression in a single lineage i.e. erythroid cells during terminal differentiation [13,14]. Whether enhancers act redundantly or in a functionally specific fashion remains to be clarified at numerous gene loci. Importantly, several genome-wide studies demonstrated that the mammalian chromatin regulatory landscape almost invariably involves distal regulatory elements, with the median enhancer-promoter distance ranging between 80 to 120 kb [15,16,17], and reaching up to more than 1 Mb [18]. This indicates that distal regulation is the rule rather than the exception in mammalian genomes.

### 2.2. Distal Regulation by Long-Range Chromatin Contacts

A key question that arose at the time of the discovery of the globin LCR, was how distal regulatory elements could control target gene expression over such large distances. The first demonstrations that distal enhancers establish specific [19,20] and functional [21,22,23] connections, i.e. physical proximity with their target genes through chromatin looping, stemmed from the study of erythroid cells, and the adaptation of chromosome conformation capture technology originally applied on yeast genomes [24,25]. This discovery and technological advances have paved the way to hundreds of follow-up studies showing that chromatin looping is a common mechanism operating at a large number of mammalian genes [4,5,6,26,27,28,29], although other mechanisms likely exist [30]. Whereas the functional role of the various globin enhancers have been thoroughly investigated, delineating their individual contribution to globin expression, together with their dynamic long-range interactions with the globin promoters, the important question of how these elements coordinate their actions within three-dimensional chromatin structures remains poorly understood. In such chromatin configurations, are the different enhancers (comprising the LCR) looping individually and sequentially towards the globin gene promoters, or is the strong globin activation the result of increased and/or stabilized contacts of the whole LCR with the target promoters? Can several promoters under the control of shared enhancers be regulated simultaneously or in a sequential manner?

## 3. Enhancers Can Form Hubs That Can Simultaneously Accommodate Different Promoters in Higher-Order Chromatin Structures

**Figure 1:**
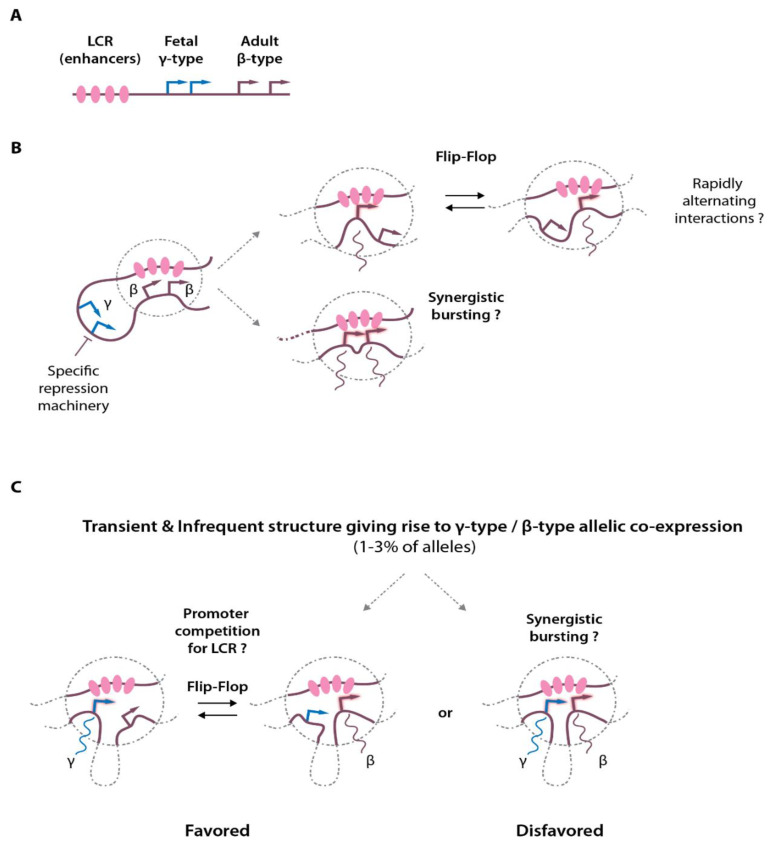
Chromatin interactions at the globin locus, multiway contacts and transcriptional activation. (**A**) Simplified scheme of the β-globin locus showing the enhancers (LCR) and the globin gene transcription start sites (arrows). (**B**) Activation of the adult-type globin genes in adult erythroid cells. Looping between the LCR and the globin promoters allows transcriptional activation that may exist in different flavors: either through rapid alternating contacts (flip-flop mechanism), or through synergistic activation (synergistic transcriptional bursting). Please note that structures containing several promoters and the LCR enhancer hub were detected by Tri-C and MC-4C. (**C**) In a small proportion of cells, concomitant expression of the fetal (γ-globin) and adult (β-globin) can be detected on the same allele. The plausible models are depicted, with the flip-flop model being statistically favored.

Because most of the current chromatin-looping assays are performed on a population of cells, it only provides an average view of the chromatin contacts, preventing dissecting chromatin contact dynamics in individual cells and on individual alleles. In the original models put forward in the 1980s and 1990s, it was proposed that the different promoters would compete for the access to the enhancers (reviewed in [31]). In this model, the expression of the different globin genes would be mutually exclusive as interaction with the LCR enhancers is needed for proper transcriptional activation. Although this is truly the case between the silenced fetal and adult promoters in adult erythroid cells [32,33], it remains unclear in situations where several active promoters within the same locus rely on common enhancers for their activity. To distinguish whether individual interactions occur simultaneously or in a mutually exclusive manner, two recent elegant studies developed novel dedicated 3C-based approaches (“Tri-C” and Multicontact 4C—“MC-4C”) to enable single-allele detection of multiway chromatin contacts [34,35]. Interestingly, these studies detected multiple simultaneous chromatin contacts between the α- and β-globin enhancers and the globin genes, showing the existence of enhancer hubs (Figure 1A,B). These studies also demonstrated that these hubs can accommodate more than one promoter with the detection of both active adult β-globin gene promoters in close contact with the enhancers. However, it appears clearly that the developmentally silenced fetal globin genes remain mostly excluded from this chromatin topology, as they are subjected to specific repression in adult erythroid cells [33,34,35,36] (see Section 6), although both fetal and adult globin genes may show concomitant expression at low frequency in cell populations [32,37,38] (Figure 1B,C). 

**Figure 2:**
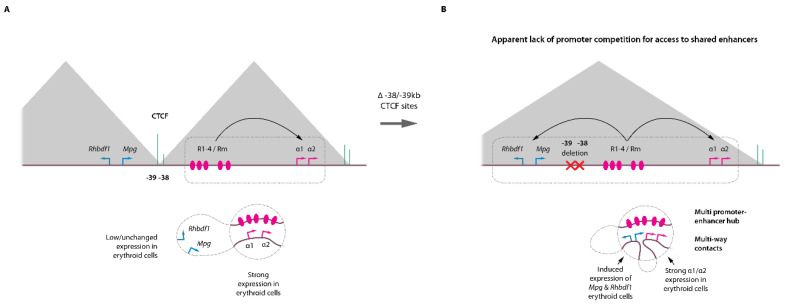
Enhancer–promoter hubs in erythroid cells. (**A**) Top, cartoon representation of the mouse α-globin locus with the corresponding (sub-)TADs (grey triangles), key CTCF binding sites (green) and α enhancers (R1-R4, Rm). Bottom, distal enhancer looping towards the α-globin promoters induce strong transcriptional activation. The −38/−39 kb CTCF elements act as a barrier to loop extrusion preventing interactions with the *Mpg* and *Rhbdf1* in the neighboring TAD. (**B**) Engineered locus with focused deletion of the CTCF −38/−39 kb sites. The absence of these CTCF elements leads to concomitant activation of the *Mpg* and *Rhbdf1* and α-globin genes by the R1-R4/Rm enhancers. The enhancer hub can accommodate several promoters in a single structure without evidence for promoter competition for access to shared enhancers.

A subsequent study applying Tri-C on erythroid cells with an extended α-globin domain further confirmed the observation that several promoters can colocalize within an enhancer hub [39]. In this model, the deletion of a few base pairs corresponding to two upstream CTCF motifs (located at −38/−39 kb) results in an expansion of the α-globin topological domain [40], with associated increased ectopic contacts of the enhancers with two upstream genes, *Mpg* and *Rhbdf1* which are normally present in the neighboring domain and which do not interact with the α enhancers in wild-type cells (Figure 2A,B). This new chromatin configuration is associated with the up-regulation of *Mpg* and *Rhbdf1* expression offering a unique opportunity to study whether promoter competition takes place within a domain where multiple promoters are regulated by a common set of enhancers. Strikingly, Tri-C highlighted the interaction hubs containing complexes of the α enhancers, the α-globin gene promoters together with the *Mpg* and *Rhbdf1* promoters. Altogether these observations indicate that individual components of an enhancer cluster as present at α- and β-globin loci can therefore spatially aggregate into a single structure that can accommodate several promoters simultaneously for transcriptional activation. As these structures are likely highly dynamic [41,42] and present at relatively low frequencies, they were missed in conventional population-based assays. Importantly, such structures were also observed on the *Pcdhα* protocadherin gene cluster in fetal brain neurons, showing that these concepts are not specific to the globin genes or to erythroid cells, but represent basic general mechanisms operating at different loci in different tissues [34]. 

It should be noted, however, that despite detecting chromatin hubs with multiple enhancers and promoters, these studies do not demonstrate that the various gene promoters present within these hubs display synchronized induction of transcriptional bursts (Figure 1B). It remains possible that genes within these hubs alternate very rapidly to establish sequential productive interactions with the enhancers for their differential activation. One technical limitation is that 3C-based assays rely on chromatin crosslinking. The kinetics of crosslinking may allow rapidly alternating chromatin structures to be captured within the same structure over the time window used for efficient crosslinking (minutes), therefore blurring the picture and giving rise to such enhancer–promoter clusters. Validating these results with orthogonal assays relying on non-crosslinked material or high-resolution (live) imaging will be essential when technologies are mature enough to visualize such small and dynamic structures. All considerations taken apart, given the recent discovery that the nuclear environment is mostly repressive with chromatin repressive complex components largely exceeding co-activator proteins [43], it is tempting to speculate that such enhancer hubs may represent an efficient way to attract and cluster co-activators in order to create an increased local concentration of these molecules, perhaps into the form of phase-separated biomolecular condensates [44], for efficient transcriptional activation.

## 4. Enhancers-Promoter Contacts: Are They Both Static and Dynamic Structures?

It should be noted that although long-range enhancer–promoter chromatin interactions are highly dynamic [26,41,45,46], the precise nature of chromosomal organization during active transcription remains poorly characterized. Precisely time-resolved 3D contact measurements at model loci (e.g., *Kit*, *CD47*) have revealed that distal enhancer–promoter interaction can form stable chromatin complexes that engage into the gene body during productive transcriptional elongation, following the edge of elongating RNA polymerase II [47]. Enhancer–promoter contacts measured by conventional 3C in this study appeared stable during the entire transcription cycle (~30 min), whereas this structure showed dynamic interactions within the gene body, matching progression of the elongation and transcription machinery. However, it remains to be understood how this model applies to multi-gene loci such as the globin loci where the enhancer hubs can accommodate multiple promoters in a single structure [34,35], or in the case of intragenic (i.e., intronic) enhancers. On the other hand, other reports detected stable chromatin contacts with pre-looped structures that were independent on the transcriptional status of the target genes [48,49,50,51]. One model put forward is that such stable and pre-established enhancer–promoter interactions may represent an efficient way to prioritize expression of subsets of genes upon a given stimulus [50]. This may explain why common extracellular signals such as TNF-α, IFN-γ, β-estradiol may elicit very different responses in different cell types while activating similar intracellular machineries.

## 5. Mechanistic Insights into Chromatin Loop Formation and Function

### 5.1. Nuclear Proteins Involved in Chromatin Looping

**Figure 3:**
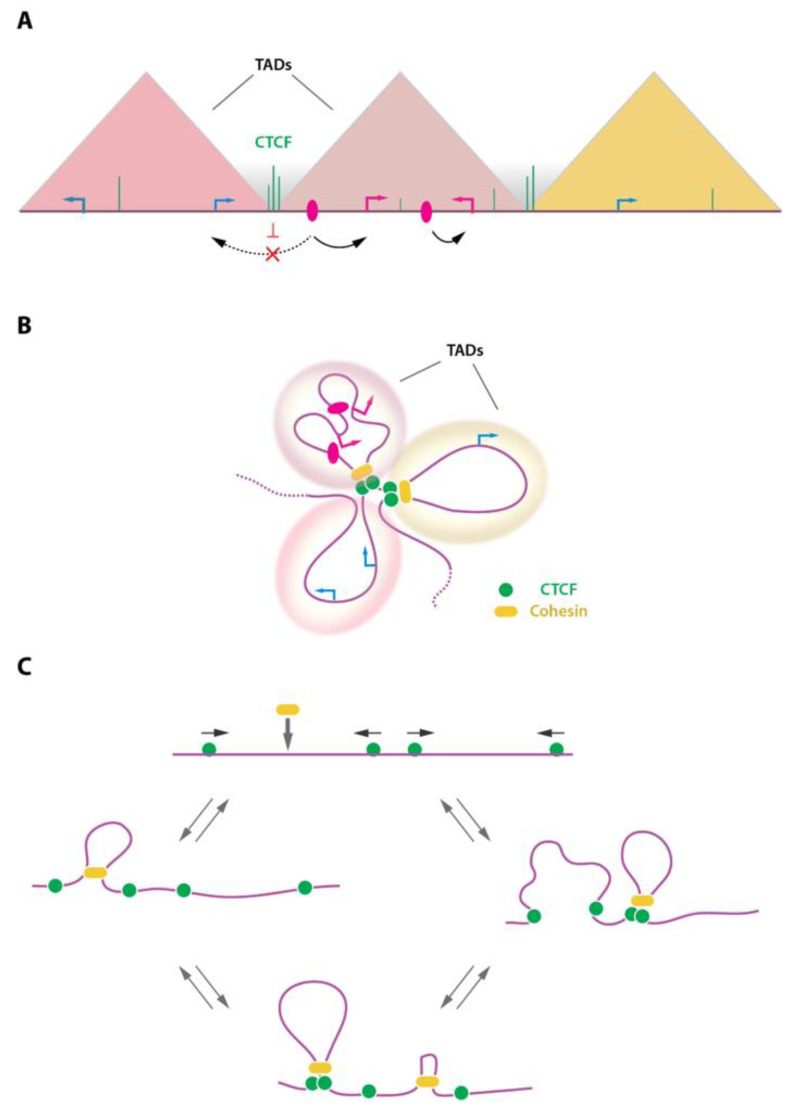
TADs and the loop extrusion process. (**A**) Schematic representation of TADs (triangles) as observed in Hi-C contact maps. TADs represent domains of favored chromatin interactions. As a consequence, enhancer (pink ovals)-promoter interactions are favored within TADs (black arrows) and disfavored across TADs (dashed arrow). TADs are separated by borders, often enriched in CTCF binding sites, which may contribute to prevent interactions between domains. (**B**) TADs representation as folded chromatin. TADs are formed and stabilized by the action of CTCF and the cohesin complex. (**C**) The loop extrusion model is depicted showing recruitment of the ring-shaped cohesin complex onto the genome where it initiates DNA extrusion, forming a chromatin loop. The cohesin complex stalls are convergently oriented CTCF sites. This process is dynamic and assembles/disassembles continuously as a function of CTCF and cohesin residence time on chromatin. Arrows on top of CTCF (green circles) indicate the orientation of the underlying DNA motif.

Different levels of chromatin loop or domain formation co-exist in mammalian cells. At the tens to hundreds kilobases scale, chromosomes are folded into so-called Topologically Associating Domains (TADs). The TADs partition our chromosomes and are believed to be involved in the insulation of gene expression and to prevent, to some extent, uncontrolled enhancer promoter communications (Figure 3A,B). In the current models, TADs are formed by a dynamic process known as ‘loop extrusion’ whereby the ring-shaped cohesin complex is loaded on the DNA and progresses along the chromatin fiber (reviewed in [6,26,52]) (Figure 3C). This continuous process leads to an active engulfment of chromatin into the cohesin ring, forming a chromatin loop which progressively increases in size until the cohesin complex stalls at convergently oriented CTCF-bound sites [16,53]. Transient stabilization of such structures gives the characteristic TAD signals and loop domains typically observed in Hi-C whole-genome 3D chromatin maps. It also matches the position of TAD borders, which are believed to prevent communications between regulatory elements and genes belonging to different TADs, and to rather favor intra-TAD chromatin interactions (Figure 3). It should be noted that cohesin can also accumulate at sites devoid of CTCF, including at MCM (Minichromosome maintenance) complex binding sites [54], or by RNA polymerase II at some gene promoters [55,56] and/or at G-rich sequences able to form non-canonical DNA structures such as G-quadruplexes [57]. At the intra-TAD level, chromatin looping favors long-range enhancer-promoter communications at the basis of gene expression regulation. It is believed that the loop extrusion process by the cohesin complex provides a framework to favor such chromatin-chromatin communication within TADs, while disfavoring interactions across borders.

**Figure 4:**
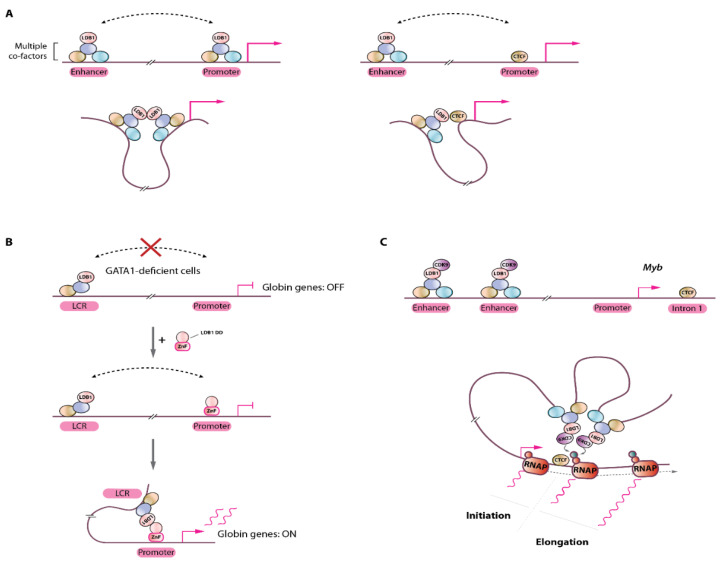
Chromatin loop formation by the LDB1 transcription factor. (**A**) Left, distally-bound LDB1-complex (containing several DNA-binding TFs and co-factors) at enhancers can interact with promoter-bound complexes by self-interactions (via its homodimerization domain); Right, LDB1 can also interact with promoter-bound CTCF through heterodimerization, bringing enhancer sequences to close proximity to promoters for efficient transcriptional activation. (**B**) in GATA1-deficient cells, the LDB1 complex does not bind the globin gene promoter but interacts with the LCR enhancers (via the TAL1 TF). Note that LDB1 does not directly bind DNA. In this context, chromatin looping does not occur and the globin genes are silent/poorly activated. Artificial tethering of the LDB1 dimerization domain (DD) through fusion to a Zinc-finger (ZnF) domain engineered to bind the globin promoter sequence results in re-establishment of long-range interaction with the distal LCR and activation of globin genes transcription. (**C**) Schematic representation of the *Myb* locus with LDB1-complex bound distal enhancers. Recruitment of the CDK9 kinase at enhancer sites by the LDB1-complex stimulates transcription elongation at *Myb* intron 1 by looping through local phosphorylation of RNA polymerase II (RNAP). Red and green circles on RNAP: Ser5- or Ser2-phosphorylated RNAP C-terminal domain, respectively.

Besides the clear roles played by CTCF and the cohesin complex which have been thoroughly investigated in the context of higher order 3D genome structures, functional enhancer-promoter communications within such domains were shown to require TF and other types of chromatin complexes. Within these permissive structures, functional enhancer-gene communications take place via protein-protein interactions stabilizing interactions between regulatory elements and their target genes. These so-called “looping factors” mostly belong to the TF category (e.g., LDB1, ZNF143, YY1, the Mediator complex) [21,58,59,60]. Besides CTCF which also plays a role in functional enhancer-promoter communications, LDB1 (LIM-Domain Binding Protein 1) is to date the best characterized chromatin looping factor [4,5,21,23,46,61,62] (Figure 4A). LDB1, a ubiquitously expressed and essential gene [63], is engaged in a multiprotein complex composed of the essential proteins GATA1, TAL1, ETO2/CBFA2T3, LMO2, E2A, and many other factors (e.g., IRF2BP2, NCOR1, LSD1) in erythroid cells [62,64,65]. LDB1 and its drosophila ortholog Chip were shown to facilitate or be required for chromatin looping [46,62,66,67,68], however precise mechanistic insights were lacking. An elegant study using the β-globin locus as model subsequently dissected the molecular mechanisms at play and demonstrated that LDB1, through its homo-dimerization domain was able to bring together distally bound genomic elements through a self-interaction process [21] (Figure 4A,B). Follow-up studies using in depth biochemical characterizations provided a structural framework for LDB1-mediated interactions, and suggested that (part of) the mechanisms are evolutionarily conserved up to the plant kingdom [69]. Interestingly, the YY1 TF was also shown to act as a looping facilitator through its homodimerization [60], suggesting that self-interaction domains represent an important and efficient mean to organize complex 3D genome structures. It should be noted that additional types of interactions are involved, as heterodimerization between distally-bound LDB1 and promoter-bound CTCF were also shown to mediate chromatin looping, primarily happening at erythroid genes throughout differentiation [70]. Importantly, the combined capacity of LDB1 to establish long-range chromatin communications and to act as a recruitment platform for several transcriptional co-factors potentiates the action of enhancers, culminating in robust transcriptional activation [23]. The mechanisms by which other types of chromatin looping factors such as ZNF143 regulate chromatin interactions to enable precise control of transcription remain to be established but they very likely involve interactions with distally bound co-factors, or with large chromatin complexes, such as Mediator, bridging distally-bound regulatory elements with promoters.

### 5.2. Functionality of Chromatin Looping

An important remaining open question concerns the functional role of chromatin loops. Are distal enhancer-promoter loops really important and how do they mediate their role? Are they fulfilling an instructive role (i.e. impacting on the transcription cycle) or are they only a consequence of transcriptional activation, i.e. being permissive structures that are formed following transcriptional activation? A key answer to these questions was provided by artificial tethering of LDB1 to the globin locus, showing that in the absence of GATA1, forced localization of the LDB1 dimerization domain at the β-globin gene promoter induces globin expression, a phenomenon depending on chromatin looping with the upstream LCR [21] (Figure 4B). Therefore, in this system, LDB1-mediated looping has an instructive role leading to robust transcriptional activation. This was further confirmed when LDB1 was artificially targeted to the silent fetal globin gene promoter, thereby creating de novo loop formation with the upstream LCR, leading to reactivation of this developmentally silenced gene with a reciprocal reduction in adult type globin gene as the LCR establishes new contacts primarily at the fetal gene [22].

A function for LDB1-mediated loops in the stimulation of transcriptional elongation was suggested at the *Myb* gene locus, where it was shown that LDB1 recruits the CDK9 kinase at the distal enhancers which would phosphorylate RNA polymerase II serine 2 (a mark of productive elongation) via looping [46,64]. The main enhancer looping sites were clustered on the *Myb* gene first intron, precisely at the site of the transition between transcription initiation to elongation (Figure 4C). In the presence of CDK9 inhibitor DRB, loops between the distal enhancers and the *Myb* gene were maintained but failed to activate *Myb* transcription [46]. These observations indicate that looping is not dependent on active transcriptional elongation (as was shown in other studies [47]), and suggest that at least one function of chromatin loops may be to stimulate transcriptional elongation via long-range chromatin contacts, although distal enhancers may likely influence different steps of the transcription cycle, including transcription initiation, depending on the target gene considered [71].

Increased proximity between regulatory elements and genes is a common feature underlying transcriptional activation as observed on numerous gene loci. However, it is noteworthy that the opposite was also observed: increased enhancer-promoter distance was recently demonstrated at the *Shh* locus in embryonic stem cells differentiating to neural progenitors [30], correlating with gene activation. The mechanistic bases are not clear but it was suggested that increased spacing between enhancer-promoter pairs in this context requires the enzymatic activity of the poly(ADP-ribose) polymerase PARP1, plausibly establishing of a molecular meshwork functionally relaying enhancer activity onto the target gene. The observed increased distance are therefore incompatible with direct looping mechanisms, and strongly suggests that additional mechanisms also take place in mammalian cells.

In aggregate, these observations suggest that multiple regulatory components control long-range chromatin interactions for the fine-tuning of transcription. As technologies allowing high-resolution chromatin contacts measurements, and enabling precise manipulation and tracking of candidate factors progress at an incredible pace [72,73,74,75,76,77,78,79,80], it is likely that additional components and novel mechanistic insights of this sophisticated process will come to light in the near future. 

## 6. Unraveling the Globin Regulatory Network: Novel Players in a Crowded Field

The β-globin gene locus has long been used as a model to dissect chromatin-looping dynamics and developmental gene regulation, and it continues to be a widely studied locus [71,72,81,82]. This high interest in globin gene loci stemmed in part from the observation that in β-thalassemia patients lacking sufficient expression of the adult-type β-globin gene, the induced reactivation of the silent fetal globin genes (γ-globin) could attenuate disease severity by substituting for the missing β-globin and assembling into functional hemoglobin molecules, rescuing erythroid differentiation. Therefore, the globin gene loci served as models for developmental gene switching and attracted a lot of attention. First insights into the mechanisms of fetal globin repression mechanisms originally came from large-scale genome-wide association studies in humans. By studying individuals with natural persistence of fetal hemoglobin in adulthood, three loci were found to be genetically linked to fetal globin repression: the globin locus itself, and the *BCL11A* and *HBS1L-MYB* loci. This led to the identification of the BCL11A TF as a potent direct repressor of γ-globin through binding the promoter and recruiting repressive components of the CHD4/NuRD chromatin-modifying complex [83], also acting as a competitive inhibitor of NF-Y co-activator recruitment on the γ-globin promoter [84]. Further human genetic analyses identified the KLF1 and MYB TFs as potent regulators of γ-globin repression, as decreased *MYB* expression and *KLF1* haploinsufficiency in humans associate with hereditary persistence of fetal hemoglobin [68,85,86]. As is also the case for MYB [68], KLF1 seems to be involved in the regulation of the major γ-globin repressors (e.g., BCL11A). During recent years, an increasing number of direct and indirect γ-globin repression mechanisms have been identified (reviewed in [36]), including additional TFs (SOX6, ETO2/CBFA2T3, LRF/ZBTB7A, etc.), and chromatin-modifying enzymes such as the LSD1 H3K4 demethylase. Interestingly, recent Crispr-Cas9 genetic screens also highlighted novel γ-globin repression machinery components. A kinase domain-focused screen using improved guide RNA scaffold identified the HRI kinase as potent γ-globin repressor, controlling transcriptional activation of the *BCL11A* gene [87]. Interestingly, HRI is known to act as a sensor of excess heme therefore fine-tuning translational activity to maintain proper heme/globin balance during terminal erythroid differentiation [88]. Although HRI is known to selectively regulate cellular protein translation through phosphorylation of the translation initiation factor eIF2α, the mechanistic links between HRI and transcriptional control of *BCL11A* remain unclear. 

### The NuRD-ZNF410 Axis in Fetal Globin Control

Interestingly, by harnessing the power of Crispr-Cas9, an elegant study using in situ saturated mutagenesis have delineated the specific residues (protein interfaces) within NuRD complex components involved in γ-globin repression (e.g., CHD4-GATAD2A) [89]. Although CHD4 knock-down/knock-out is deleterious for erythroid cells, key protein residues were identified in defined protein domains that could be mutated without altering cellular fitness while impacting fetal globin genes repression, offering opportunities for therapeutic intervention [89]. Subsequently, recent work using a TF DNA-binding domain-focused CRISPR library identified ZNF410 as a new γ-globin repressor [90]. The findings reported in this study are intriguing as, contrarily to most TF, which tend to occupy thousands of genomic binding sites, ZNF410 was shown to only occupy eight loci, with only one, the *CHD4* locus, being highly enriched and functionally occupied (i.e., sensitive to ZNF410 levels). Careful control experiments were performed to ascertain that this peculiar binding pattern was a true biological observation, confirming that *CHD4* is the only direct target gene that is sensitive to ZNF410 dosage. Shortly thereafter another study reported similar findings, independently confirming this surprising observation [91]. Upon ZNF410 suppression, *CHD4* levels decrease by ~70%. As the main direct γ-globin repressors BCL11A and LRF/ZBTB7A use the CHD4/NuRD complex as a mean for repressing γ-globin genes, decreased CHD4 levels lead to γ-globin reactivation. Surprisingly, the only measurable effect of ZNF410 depletion is the reactivation of γ-globin due to CHD4 down-regulation, which happens in an otherwise non-pathological terminal differentiation context (both in primary human cells and in the mouse in vivo) [90,91]. The clusters of ZNF410 DNA-binding motifs particularly enriched at the *CHD4* promoter and distal regulatory element, are the only regions of the entire genome with such high density of perfect consensus motifs (clusters of more than 10 motifs). Interestingly, these regions exhibit high evolutionary conservation, indicating that such sequences have been positively selected or co-opted by ZNF410 for proper functions. This may sound surprising as repression of the fetal γ-globin genes is not an absolute requirement for erythroid cell survival since γ-globin and HbF persistence has long been observed in the human population without any associated erythroid defects. However, as CHD4 has a broad expression pattern, such ZNF410 clusters of binding sites close to *CHD4* may be essential in other tissues where its function may be limiting. It should be noted that one of the studies report that *Zfp410* (the murine *ZNF410* ortholog) KO mice show no overt abnormalities but littermate have slightly decreased body weight, indicating that ZNF410-mediated control of CHD4 may be important during specific developmental windows or within specific contexts that remain to be determined [91].

Besides the role of ZNF410 in HbF silencing, this study provides one of the very few examples of mammalian TF with an extremely small number of binding sites throughout the genome (in that case less than 10, with functional impact on only one gene, *CHD4*, in erythroid cells). The mammalian regulatory landscape therefore involves atypical TF binding events, with extremely restricted functional consequences [92]. As ZNF410 inactivation has no obvious phenotypic impact on erythroid cell differentiation, both on cell lines, KO mice or primary human cells, it would not score as an essential erythroid regulator in conventional drop-out screens, despite its key role on γ-globin repression (through *CHD4* regulation). This result underscores the usefulness of carefully designed Crispr screens with clear phenotypic readout to uncover novel regulatory mechanisms. These studies further highlight how intensive continuous research focused on deciphering the repressive mechanisms occurring at fetal globin genes has uncovered surprising and novel biological features. Whether such a peculiar low binding site TF is actually a widespread feature of gene networks in hematopoietic cells and other tissues remains to be uncovered, but these findings further expand the characterization of the regulatory networks at play in erythroid cells, and provide a unique framework for integrative analyses of cellular differentiation, and interplay between TF activity and coordinated activation/repression of gene networks.

## 7. Systems Approaches to Dissect Erythropoiesis: New Discoveries and Former Concepts Revisited from Proteomic Studies

Single-cell analyses have greatly contributed to our understanding of erythroid commitment and provided a refined understanding of the hematopoietic differentiation tree [93,94,95,96,97,98]. These studies provided a systems approach to dissect erythropoiesis and have been an invaluable source of discoveries. However, although measurements of transcript abundance is often used as a surrogate for protein expression, several proteomic analyses of erythroid cells have shown that RNA levels may correlate very poorly with protein levels [43,99,100,101], emphasizing the importance of direct protein quantifications.

Importantly, proteomic quantification of the erythroid proteome combined with mathematical modeling provided recent important insights into the regulatory networks at play during erythropoiesis [43]. Erythropoiesis is under the control of several lineage-specific as well as general TFs that establish and orchestrate stage-specific chromatin environments. Engagement towards the erythroid lineage during hematopoietic stem cell differentiation is the net result of the counteracting actions between myelo-erythroid TFs favoring different cell fates [96]. This is best exemplified by the antagonistic action of myeloid-driving TFs such as PU.1 and erythroid-driving TF GATA1, or between the early hematopoietic regulator GATA2, which controls HSCs and early immature progenitor fates, and GATA1 which becomes activated during erythroid differentiation and counteracts GATA2 activity leading to robust erythroid lineage commitment, a process known as the “GATA-switch”. 

### 7.1. GATA-Switch Revisited

The above-mentioned process of GATA2-to-GATA1 expression transition is known as the “GATA-switch” whereby GATA2 activates *Gata1* gene expression in immature progenitors and in turn GATA1 represses *Gata2* expression allowing cells to acquire an erythroid fate (reviewed in [102,103]). This type of cross-control by lineage-specifying master regulators has been widely studied and served as a paradigm for epigenetic mechanisms and cell fate commitment. The original model proposed that GATA2 was progressively displaced from its binding sites by increasing GATA1 concentrations (and progressively decreased GATA2 levels as GATA1 represses the *Gata2* locus). However, a recent study revealed that contrarily to what was originally thought, the levels of GATA1 largely exceed the ones of GATA2 already from the earliest CFU-E onwards. This implies that GATA2 displacement from its chromatin binding site is not the mere reflection of GATA1 versus GATA2 protein dosage, and suggests that GATA2 can maintain its genomic binding even in the presence of an excess of GATA1. This indicates that dynamic GATA2 occupancy during erythropoiesis is more likely the result of decreased GATA2 concentration in differentiating erythroid progenitors rather than a direct displacement by excess GATA1. This switch is further reinforced by the action of other TFs such as TAL1, which activates *Gata1* more potently than GATA2 does, probably because TAL1 protein levels were shown to be on average three times more abundant than GATA2. In light of these novel discoveries, the GATA-switch model has been refined and it opens up new questions such as how GATA2 and GATA1 co-exist and maintain their respective binding sites in the genome, and sheds new light on the combined actions of synergistic and antagonistic TFs. The mechanistic bases of the GATA-switch remain incompletely understood. A possible explanation for the capacity of GATA2 to maintain its genomic binding sites without overt displacement in the presence of its repressor GATA1 could be the result of a gradual capacity of GATA1 to occupy its genomic target sites, or an increased off rate of GATA1 at the early stages of erythropoiesis. This way, if GATA1 chromatin binding is not fully stabilized in immature progenitors, it would leave GATA2 with the possibility to properly regulate its target genes even in the presence of excess GATA1. Testing this hypothesis requires further quantitative measurements of relative dynamic chromatin occupancy. The advances in single-molecule tracking may offer new opportunities to shed light on this intriguing question [78,79,80]. It would also be important to test whether the observed role of GATA1 as a chromatin bookmarking factor during cell division [104] further impacts GATA2 activity, and whether GATA1 bookmarking is already active in the immature progenitors compartment where GATA1 is already abundant or whether it becomes active at the onset of erythroid terminal differentiation of pro-erythroblasts (correlating with GATA2 displacement and decreased activity). This type of TF network controlling the progression of a specific lineage is likely applicable to other hematopoietic and non-hematopoietic lineages and provides a framework for better understanding gene regulatory networks operating during cellular differentiation.

### 7.2. Myeloid TF Expression in Erythroid Cells and Lineage Restriction

Several TF related to HSC maintenance (e.g., RUNX1) or belonging to other myeloid lineages (e.g., FLI1, CEBP-β) continue to be expressed in lineage-committed erythroid progenitors. Although their relative levels decline during the course of erythroid differentiation, they are still detectable up to the CFU-E stage, which suggests that committed erythroid cells may retain some degree of lineage plasticity [43,96,105]. A recent study using conditional ablation of the epigenetic regulator LSD1 (a.k.a. KDM1A) demonstrated that committed erythroid progenitors maintain the ability to differentiate into myeloid cells and that lineage restriction is actively epigenetically controlled (e.g., via LSD1) [105]. Inhibition of LSD1 activity leads erythroid progenitors to undergo myeloid differentiation, a process depending on the expression of the myeloid TFs PU.1 and RUNX1. Lineage tracing experiments using the Rosa26-TdTomato reporter showed that the increased GMP/GMP-like myeloid cells derive from TdTomato^+^ erythroid progenitors that likely undergo a lineage fate change upon Lsd1 ablation. This lineage conversion could be recapitulated in vitro upon the use of LSD1 inhibitors both on mouse and human erythroid progenitors, confirming the essential role played by LSD1 in maintaining erythroid cell identity. Gene expression analysis showed that the PU.1 TF is overexpressed in LSD1-deficient erythroid-derived myeloid cells. Mechanistically, LSD1 was shown to directly occupy the *PU.1*/*Sfpi1* gene transcription start site and to maintain low H3K4 methylation modification, contributing to transcriptional repression. The RUNX1 transcription factor, which is a known positive regulator of PU.1 expression in stem cells and myeloid cells, contributes to PU.1 reactivation upon LSD1 loss of activity, and the erythroid-to-myeloid lineage transition could be rescued by inactivating either PU.1 or RUNX1 in Lsd1−/− cells. This confirms that a RUNX1/PU.1 axis is reactivated upon loss of LSD1 activity, which confers the ability to counteract the erythroid program and instruct a myeloid transcriptional state, at the origin of the observed erythroid-myeloid lineage plasticity [105].

This is a surprising result as it shows that committed erythroid progenitors retain the capacity to diverge into an alternative myeloid fate, and that hematopoietic lineage restriction is relying on an active epigenetic mechanism to suppress alternative (myeloid) lineages, rather than being directed by a unidirectional and irreversible engagement into lineage-specific paths. The persistent (moderate) expression of myeloid TFs in erythroid progenitors (CFU-E) is key to this lineage plasticity and supports the idea that hematopoietic differentiation should be considered to be a continuum of cell states with antagonistic epigenetic switches favoring one lineage over another. However, whereas the erythroid progenitor-derived myeloid cells in *Lsd1* KO animals express myeloid differentiation markers (Gr1^+^, CD11b^+^) [105], it is not known whether these are fully functional (i.e., fully converted) or whether conflicting transcriptional programs co-exist in these cells.

## 8. Signaling Pathways Controlling Erythropoiesis: Old Players, New Targets

**Figure 5:**
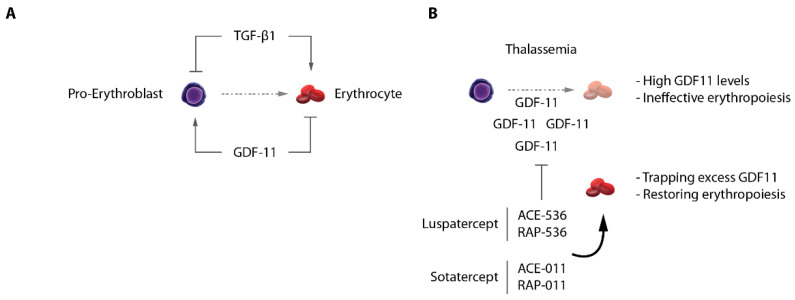
TGF-β/GDF-11 signaling in erythropoiesis. (**A**) TGF-β1 and GDF-11 have opposite roles on terminal erythroid differentiation. (**B**) In β-thalassemia, excess GDF-11 acts as an autocrine inhibitor of differentiation, being a cause of ineffective erythropoiesis. Ligand traps Luspatercept and Sotatercept normalize GDF11 levels in vivo and significantly restore erythroid differentiation.

Erythropoiesis is under the control of key signaling pathways regulating progenitor proliferation, survival and differentiation. Besides the well-known roles of SCF/c-KIT signaling to sustain immature progenitors proliferation, and the well-established role of the EPO/EPOR axis [106], and glucocorticoids [107] in erythroblasts survival and maturation, additional pathways were shown to have strong impacts on red cell production. The TGF-β pathway for instance has a strong pro-differentiation action on erythroid progenitors. It was shown that when cultured in the presence of TGF-β1, primary human progenitors undergo a massive proliferation arrest coupled to the entry into terminal differentiation [108,109] (Figure 5A). A more recent study combining profiling of the erythroid proteome with focused CRISPR screen confirmed the importance of this pathway through the identification of the TGF-β receptor TGFBR2 as a key effector of terminal differentiation [110]. This indicates that one role of the TGF-β pathway is to accelerate erythroid differentiation, at the expense of immature progenitor proliferation, hence limiting erythroid cellular output while inducing fast maturation outcome. It should be noted, however, that despite reports showing that pathways activated by extracellular signals such as TGF-β converge on chromatin sites occupied by master erythroid regulators (e.g., GATA1), and hence may modulate their activities [111,112,113], the molecular mechanisms underlying TGF-β functions in erythropoiesis remain poorly understood. Furthermore, the precise role of the TGF-β pathway, and the sources of TGF-β superfamily ligands production within the erythroid niche in vivo remain poorly characterized. One role of this pathway on erythroid cells may be to restrict and/or to fine-tune the daily production of erythroid cells in the body. The demonstration of the clinical importance of TGF-β signaling in erythroid disorders in vivo came from the discovery that ineffective erythropoiesis in β-thalassemia is to a large extent the result of aberrant TGF-β superfamily signaling [114,115]. It was shown that in mouse models of thalassemia, extramedullary splenic stress erythropoiesis is associated with overexpression of the TGF-β superfamily ligand GDF11. GDF11 is produced by immature erythroblasts, probably as a consequence of increased oxidative stress in thalassemic erythroblasts, and acts in an autocrine fashion to maintain progenitor proliferation and inhibit terminal differentiation (Figure 5A). Increased serum GDF11 levels were also detected in thalassemia patients, confirming the data obtained in mice. Importantly, the use of the activin receptor ligand traps Sotatercept (ACE-011) or ACE-536 in humans, or RAP-011 (the mouse equivalent) leads to efficient inhibition of GDF11 signaling, and restoration of hematologic parameters, suggesting that GDF11 is a major component of the ineffective erythropoiesis in thalassemia. This important discovery of the TGF-β1/GDF11 imbalance impairing erythroid differentiation in thalassemic models represents a major therapeutic opportunity that led to FDA and EMEA approval of the Luspatercept, a trap receptor of GDF-11 [116,117] (Figure 5B). Surprisingly, GDF11-induced signaling in erythroblasts activates the SMAD2/3 intracellular effectors, similar to TGF-β1, whereas the effects mediated by those two ligands result in completely opposing outputs (i.e., proliferation of progenitors and inhibition of terminal differentiation by GDF11 as opposed to decreased progenitor proliferation and accelerated differentiation by TGF-β1). The molecular mechanisms acting downstream of GDF11/TGF-β1 remain unclear and warrant further investigation.

## 9. Erythroid Master Regulator Dysfunctions in Erythroid Disorders and Erythroid Leukemia: A Focus on GATA1

### 9.1. Genetic Erythroid Disorders Directly and Indirectly Converge on GATA1 Activity: Examples of Congenital Anemias and Diamond-Blackfan Anemia

**Figure 6:**
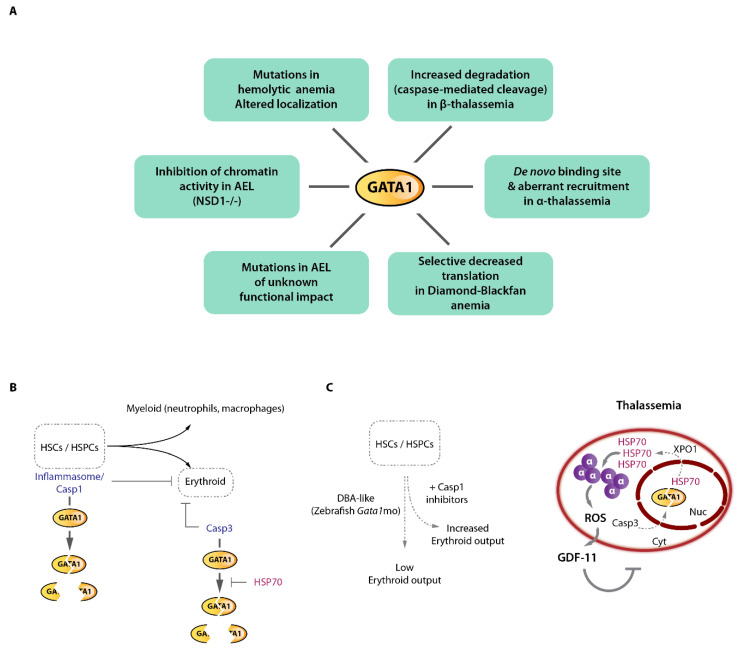
GATA1 dysfunction in erythroid disorders and GATA1 cleavage by caspases. (**A**) Reported mechanisms of direct GATA1 alterations or functional impairment in various erythroid disorders including acute erythroid leukemia (AEL). (**B**) GATA1 can be functionally inactivated by caspase cleavage at various stages of hematopoiesis. Inflammasome/Caspase 1 cleavage of GATA1 in hematopoietic stem cells (HSCs) and early progenitor cells (HSPCs) regulate the myeloid/erythroid balance. In erythroid committed cells, Caspase 3 cleavage of GATA1 regulate erythroid output. The HSP70 chaperone shields GATA1 from Caspase 3 proteolytic cleavage. (**C**) Left, modeling Diamond-Blackfan anemia (DBA)-like condition in zebrafish using *Gata1* morpholino (mo) shows that Casp1 inhibition partially rescued the erythroid phenotype. Right, β-thalassemia associates with excess α-globin chains which precipitate, generate oxidative stress leading to GDF11 production and ineffective erythropoiesis. The massive α-globin aggregates trap the HSP70 chaperone which exits the nucleus in an exportin 1 (XPO1)-dependent fashion, leaving GATA1 unprotected and sensitive to Casp3 cleavage, thereby reinforcing the β-thalassemia associated ineffective erythropoiesis.

As mentioned above, the GATA1 TF is a master regulator of erythropoiesis [118]. It binds thousands of erythroid enhancers and promoter elements and directly regulates the erythroid transcriptome, including the α- and β-globin gene clusters [62,119,120,121,122,123,124]. It is, therefore, not a surprise that many pathological conditions were associated with either defective or aberrant GATA1 activity (Figure 6A). Mutations in GATA1 have been associated with various types of anemias through perturbation of its DNA-binding capacity or its association with critical co-factors such as FOG1, leading to dysregulated gene expression and broad erythroid defects (nicely reviewed in [125]). More recently, rare forms of hemolytic anemia were characterized and shown to be associated with GATA1 mutations in a poorly characterized intrinsically disordered region of the protein (R307C/H), influencing its nuclear-cytoplasmic localization leading to altered chromatin localization misregulating erythroid gene expression (although the complete spectrum of defects associated with this mutation remains to be determined) [126]. The congenital Diamond–Blackfan anemia (DBA), on the other hand is a rare bone marrow failure condition associated with ineffective erythropoiesis. A majority of patient harbor heterozygous mutations in one of the 18 ribosomal proteins frequently altered in DBA, making this disease a ribosomopathy. Why mutations in ubiquitously expressed ribosomal proteins lead to erythroid-specific defects remains incompletely understood. A decrease in ribosome abundance was observed in DBA cells, which led to selective decrease of translation efficiency of ~500 mRNAs, including *GATA1* mRNA and others involved in the regulation of *GATA1* mRNA translation (e.g., RNH1). The selective decrease of GATA1 translation efficiency was explained by the peculiar 5′UTR structure of its mRNA, which makes it unique among hematopoietic master regulators [95,127]. Accordingly, translation efficiency of other hematopoietic TFs mRNAs such as *KLF1*, *MYB*, *GATA2*, *LMO2*, etc. was insensitive to decreased ribosomal proteins and ribosome content, providing a molecular explanation of the selective impairment of the erythroid lineage in DBA. Importantly, the erythroid defects could partially be rescued by raising GATA1 levels in DBA cells, underscoring the critical role played by GATA1 in this human disease. Importantly, it was shown that additional mechanisms contribute to disease severity [128] and GATA1 dysfunction in DBA. These include inflammasome-mediated GATA1 cleavage [129], and chaperone-mediated GATA1 stabilization, suggesting that several aspects of DBA critically converge on GATA1 activity [130,131] (Figure 6B,C).

### 9.2. A Single Base-Pair Change Causes α-Thalassemia through De Novo GATA1 Binding and Alteration of Chromatin Looping

**Figure 7:**
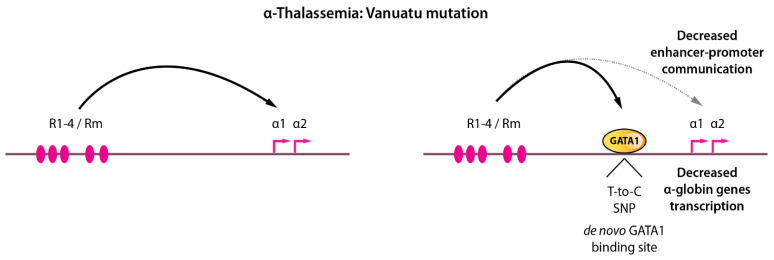
A single base-pair change in a non-coding region leads to α-thalassemia by modifying long-range enhancer-promoter contacts. Left, the α-globin locus is depicted with enhancer-promoter communications. Right, a single T-to-C single nucleotide polymorphism creates a de novo GATA1 binding motif, GATA1 recruitment, and partial rewiring of long-range chromatin contacts leading to decreased α-globin expression resulting in thalassemia.

These above-mentioned GATA1 mutations or altered levels of expression associate with widespread defects in erythroid genes expression and impaired erythropoiesis. Surprisingly, more subtle alterations were recently reported in α-thalassemic patients. The α-thalassemic condition refers to a variety of genetic defects altering α-globin genes expression, leading to globin chains imbalance, cellular toxicity and ineffective erythropoiesis. Interesting cases of α-thalassemia from Vanuatu in Melanesia [132] were reported to be associated with a single-nucleotide mutation (a T-to-C transition) in an intergenic region lying between the α-globin genes and their upstream regulatory elements (Figure 7). An elegant study recently provided an in-depth characterization of the phenotypic effects of this mutation on α-globin genes transcription [133]. Induced pluripotent stem cells (iPSCs) differentiated along the erythroid pathway were used to create (from wild-type iPSCs) or correct the mutant C-allele (from patient-derived iPSCs) by genome editing to formally prove the causative effect of this variant on the down-regulation of α-globin and defective terminal erythroid differentiation (i.e., α-thalassemia). The C-allele creates a de novo binding site for GATA1 (TTAT**T** → TTAT**C**) generating a novel transcriptionally active element in the α-globin locus, with chromatin signature matching classical promoter signatures (chromatin accessibility, H3K4me3, H3K27Ac, RNA polymerase II occupancy). It was shown that this element partially acts as an enhancer blocker, therefore “attracting” the α-globin enhancers resulting in reduced α-globin gene interactions with their native enhancers. Since the α enhancers interact more frequently with the new promoter-like element and less frequently with the native α-globin promoters, it decreases α-globin production which drives globin chains imbalance and a thalassemic phenotype. This single DNA base change therefore imposes a strong novel regulatory constraint within the locus and partially displaces long-range interactions. A surprising observation that came from this study is that this new promoter-like element acts in an orientation-dependent fashion. This implies that the enhancer-blocking activity of such element is constrained by its orientation. Although no de novo CTCF binding could be detected that could explain this orientation-dependence (as CTCF motif orientation plays key roles in organizing chromatin interactions [16,53,134,135]), it remains possible that additional as-yet-unidentified factors are recruited by GATA1 and other master erythroid TFs at this new element which may function in an orientation-dependent manner. It is not known, for instance, whether LDB1 chromatin looping is an orientation-dependent process.

### 9.3. GATA1 Cleavage by Caspases and the Protective Role of the HSP70 Chaperone in Physiological and Pathological Erythropoiesis

The importance of GATA1 in the context of thalassemia has also been underscored by the discovery that the globin chain imbalance, which is characteristic of this disorder, indirectly influences GATA1 activity [136]. Landmark studies have demonstrated that GATA1 is subjected to caspase-mediated cleavage in immature erythroblasts upon activation of the CD95/Fas death receptor [137,138] (Figure 6B). This cleavage leads to blockade of erythroid terminal differentiation as a result of defective GATA1 activity. This regulatory link is part of a feedback loop originating from maturing erythroblasts which express death receptor ligands [137]. Production of such ligands by terminally differentiating erythroblasts may inform immature erythroblasts cells of the presence of sufficient maturing cells, a process that could fine-tune erythroid cell production. During differentiation, the chaperone protein HSP70 protects GATA-1 from caspase 3-mediated cleavage, through protein-protein association [138]. Importantly, HSP70 can shuttle between the nuclear and cytoplasmic compartments [136,138,139], and upon erythropoietin starvation, HSP70 is exported from the nucleus leaving GATA1 exposed to Caspase-3 activity, resulting in differentiation arrest and apoptosis. These major discoveries led to important mechanistic insights into thalassemia-associated ineffective erythropoiesis: an elegant study demonstrated that in β-thalassemia, the cytoplasmic accumulation of the free α-globin chains resulting from the globin chain imbalance result in toxic aggregates that are taken care by dedicated chaperones (including HSP70) [136] and cellular machineries [140], in an attempt to refold these toxic aggregates and limit cellular damage (Figure 6C). As a result, HSP70 which was shown to interact with α-globin proteins is sequestered in the cytoplasm and as a consequence is not able to sufficiently accumulate in the nucleus to protect GATA1 against caspase-3-mediated proteolytic cleavage, leading to defective terminal erythropoiesis which could be restored by expression of a nuclear-targeted HSP70 mutant [136]. Besides providing critical novel mechanistic insights and offering novel therapeutic opportunities [139], this study illustrates how β-thalassemia which is primarily considered as a disease of the β-globin chains, should equally be considered as a disease of (excess) α-chains and therefore strategies aiming at reducing α-globin aggregates (e.g., [141]) or toxic hemoglobin intermediate products (such as excess heme e.g., [142]) will likely be of primary clinical importance [36].

### 9.4. Acute Erythroid Leukemia

Acute erythroid leukemia (AEL, also known as AML-M6 subtype) accounts for ~5% of all AMLs but it unfortunately stands out as being one of the most aggressive human blood cancers, associated with a median survival of 8-17 months [143,144]. Importantly, AEL is often secondary to other neoplasms such as myelodysplastic syndrome or previous cytotoxic/genotoxic treatments (they account for up to 30% of the secondary AML) [145]. Notably, the molecular etiology of AEL is largely unknown and there are no specific therapies. Due to its underrepresentation within overall AML cases, this disease has been rarely studied [146,147], and in-depth characterization of the AEL-associated mutations was lacking until recent studies reporting the mutational landscape of AEL [143,148,149,150]. These studies have shown that AEL is largely associated with TP53 mutations (TP53 being a regulator of physiological erythroid differentiation [151]), and mutations commonly found in other types of AML affecting the DNA methylation machinery (e.g., DNMT3A, TET2, IDH), or epigenetic regulators (e.g., KMT2A, NPM1, EZH2, cohesin subunits), and also commonly associate with aberrant expression of CBFA2T3/ETO2, a known GATA1 co-repressor [65,146,147,148,149,152].

Although some rare mutations of *GATA1* were detected in AEL cases [148,149] their functional impact was not evaluated. Interestingly, a recent study of AEL in murine models showed that erythroid leukemia develop in mice bearing inactivation mutations for the NSD1 (nuclear receptor binding SET domain protein 1) chromatin regulator [153] (Figure 6A). NSD1 is an histone 3 lysine 36 (H3K36) methyltransferase which catalyzes the mono- and di-methylation of H3K36 on chromatin. Whereas in *Nsd1*^−/−^ animals a signature of dysregulated *Gata1* target gene expression was detected, no changes in GATA1 levels could be observed. However, GATA1 chromatin occupancy was severely decreased in *Nsd1*^−/−^ erythroblasts, together with globally reduced mono-, di-, and tri-methylated H3K36. Using mass spectrometry analysis of differentially bound GATA1 co-factors, it was shown that NSD1 regulates the association of GATA1 with its negative regulator SKI and the NCoR co-repressor complex. In the absence of NSD1, SKI remains associated with GATA1 in differentiating erythroblasts, leading to impaired GATA1 chromatin binding, incapacity to regulate its target genes and impaired induction of terminal differentiation. Decreasing SKI expression in an *Nsd1*^−/−^ background could rescue erythroid differentiation, confirming that the main differentiation block was SKI-mediated impairment of GATA1 chromatin targeting and/or stabilization. It therefore appears that efficient deposition of H3K36 methylation marks on chromatin at gene bodies is a critical step for stabilization of GATA1 on its targets. This observation is in accordance with other reports showing that transcription elongation marks (i.e. H3K36me3) play critical roles in erythropoiesis and terminal erythroid differentiation [154,155,156]. Taken altogether these data indicate that impaired GATA1 activity plays a significant role in the differentiation block associated with AEL.

## 10. Conclusions

Erythroid research remains at the forefront of many research fields. The new molecular markers [110,157,158] and complex (epi-)genetic [96,98,159] and metabolic networks [160,161,162] that are continuously unraveled pave the way towards an in-depth understanding of erythropoiesis. Furthermore, the recent discovery of the molecular bases of new blood group antigens keep providing key fundamental and clinical insights into erythropoiesis and open up new avenues for the management of transfusion issues associated with rare blood group-bearing patients [163,164,165]. However, there is still quite a journey ahead of us before one can integrate all layers of regulation, from signaling to intracellular remodeling, metabolic switches and epigenetic responses, to provide a true systems view of this complex differentiation process. Collective international efforts to reach this goal will be indispensable to make it become a reality.
